# Supplementary material for: French General Practitioners’ Adaptations for Patients with Suspected COVID-19 in May 2020
Source: Int J Environ Res Public Health. 2023 Jan 19;20(3):1896. doi: 10.3390/ijerph20031896 (PMC9914740; doi:10.3390/ijerph20031896)
Supplement: Supplementary file 1 [file ijerph-20-01896-s001.zip › ijerph-2149029-supplementary.pdf]

Supplementary material Table S1. List of the members of the Assembler, Coordonner, COmprendre, Rechercher, Débattre en soins primaires) network (ACCORD) and their partners.

Members of the ACCORD (Assembler, Coordonner, COmprendre, Rechercher, Débattre en soins primaires) network	Partners of the ACCORD network who also sent the survey link:
National College of Academic GPs (CNGE)	French Society of General Medicine (SFTG)
SPP-IR association (Multidisciplinarity in Primary Care, Innovation and Research)	National College of General Practice (CMG)
French Society of General Practice (SFMG)	French Society of Documentation and Research in General Practice (SFDRMG)
National Federation of Multidisciplinary Groups (AVECSanté)	French Society of General Practice (SFMG)
Research Institute of Healthcare Centres (IJFR)	French Association of Young Researchers in General Practice (FAYR-GP)
RETINES (Risk, Epidemiology, Territory, INformation, Education in health) Unit, Côte d'Azur University	
National Association of Health Teams in Primary Care (ASALEE)	
RESPIRE project (EHESP)	
Midwifery Department of Versailles Saint Quentin University	

Supplementary material Table S2. Practice locations of the survey participants (n=3,068), compared with the general practitioners' (GPs) population in metropolitan France in 2019.

	**Responding GPs, n=3,068**	**All GPs in metropolitan France[1], n=53,339**
**French region, n (%)**		
Auvergne-Rhône-Alpes	450 (14.7)	6,774 (12.7)
Bourgogne-Franche-Comté	154 (5.0)	2,240 (4.2)
Bretagne	122 (4.0)	2,880 (5.4)
Centre-Val de Loire	96 (3.1)	1,760 (3.3)
Corse	0 (0.0)	267 (0.5)
Grand Est	224 (7.3)	4,694 (8.8)
Hauts-de-France	227 (7.4)	4,907 (9.2)
Île-de-France	433 (14.1)	8,161 (15.3)
Normandie	138 (4.5)	2,507 (4.7)
Nouvelle-Aquitaine	300 (9.8)	5,547 (10.4)
Occitanie	237 (7.7)	5,494 (10.3)
Pays de la Loire	223 (7.3)	2,934 (5.5)
Provence-Alpes-Côte d'Azur	131 (4.3)	5,174 (9.7)
Missing data	*323 (10.9)*	*None*

[1] Data from the French health insurance system (CNAMTS) (2019), see reference [17] in the manuscript.
